# Lemierre's Syndrome: A Lethal Complication of Acute Tonsillitis

**DOI:** 10.7759/cureus.30072

**Published:** 2022-10-08

**Authors:** Soumya Amarnani, Aditya Ranjan

**Affiliations:** 1 Otolaryngology - Head and Neck Surgery, Jawaharlal Nehru Medical College, Datta Meghe Institute of Medical Sciences, Wardha, IND; 2 Otolaryngology - Head and Neck Surgery, Jawaharlal Nehru Medical College, Datta Meghe Institue of Medical Sciences, Wardha, IND

**Keywords:** septic emboli, acute tonsillitis, fusobacterium, thromobophlebitis, leimerre's syndrome

## Abstract

Tonsillitis is a condition involving the infection of the lymphoid tissue of the tonsils. This pathology of the tonsils is frequently reported as a childhood illness in children of school-going age. The leading causative agent which is associated with tonsillitis is Group A and B Haemolytic Streptococcus and Staphylococcus and Haemophilus influenzae. With rapid and correct treatment and management with antibiotics and analgesics, it can be resolved, and the patient can be free of the symptoms such as sore throat, dysphagia, pain over the throat, and fever. Though in the minority of cases the diseases can progress and can result in multiple complications which sometimes can be lethal and extremely serious. These can be rheumatic fever, acute glomerulonephritis, or tonsillar cyst. One of the sporadic but equally important and grave syndromes is Lemierre's syndrome, which unfortunately has been labelled as a forgotten disease due to the development of antibiotic therapy and management of the disease. The mortality of the disease was extremely high in the pre-antibiotic era. This disease presents the following findings, such as thrombophlebitis of the internal jugular vein, which usually occurs after the presentation of pharyngeal infection. Which in later stages also gives rise to thrombi that advance and extend throughout the body, in the form of septic emboli. The important microorganism which is isolated and associated with the Lemierre's is Fusobacterium necrophorum, a strict gram-negative anaerobe. This article emphasizes and discusses the pathophysiology, and microbiology of Lemierre's syndrome. It also focuses on the clinical symptoms that include the appropriate and timely diagnosis and treatment of this deadly and fatal syndrome, together with the complications that arise with Lemierre's syndrome as the presenting problem.

## Introduction and background

Acute tonsillitis refers to the inflammation of the tonsils that involves the lymphoid tissue of palatine tonsils getting affected and inflamed or swollen. It is actually a very common and prevalent pathology. This pathology doesn't limit itself only to tonsils; it also involves a ring of lymphatic tissue inside the oral part of the pharynx which is the oropharynx. Acute tonsillitis term is applicable when the duration of infection is very short. It is mostly reported in school-going children as the completion of the development of the lymphoid ring has occurred which isn't the case in children of age one to one and a half years of age [1]. The most commonly infecting microorganism is Group A and B Hemolytic Streptococcus [2]. Other microorganisms that can be suspected as the causative agent are Staphylococcus, pneumococci, or Haemophilus influenzae. These bacteria may primarily infect tonsils or may be secondary to viral infection by rhinovirus, adenovirus, or coronavirus. The dominant symptoms of acute tonsillitis are sore throat, dysphagia, fever associated with chills and rigors varying from 38 degrees celsius to 40 degrees celsius, referred earache, and tender swelling over the anterior cervical lymph nodes. Clinical signs used for the diagnosis of acute tonsillitis are the following: the breath of the patient is fetid, there is hyperemia of pillars and soft palate, the tongue is coated, tonsils are red and swollen with yellowish spots of purulent material presenting at the opening of crypts or there can be whitish membrane on the medial surface of tonsils that can be easily wiped out and enlargement of jugulodigastric lymph nodes is also seen. 

Though acute tonsillitis subsides with the appropriate treatment but complications of the disease can be seen in rare cases. The complications that are worth noting are chronic tonsillitis with recurrent acute attacks, which arise due to incomplete treatment of the acute infection, there is development of pus in between the muscle of the pharynx known as constrictor muscle and the layer of capsule covering the tonsil which is called as peritonsillar abscess, abscess in parapharyngeal space, cervical abscess that occurs as a result of collection of pus within the jugulodigastric lymph nodes, rheumatic fever it is often seen in association with tonsillitis due to Group A beta-hemolytic streptococci, acute glomerulonephritis, subacute bacterial endocarditis, tonsillar cyst, tonsillolith, chronic tonsillitis [3,4]. One of the exceptionally critical but rare and important complications that need to be evaluated is Lemierre's syndrome which is basically presented in patients as extreme pain over the neck region and tenderness over the internal jugular vein and also presents with the features of septicemia. These clinical symptoms are attributed to the thrombophlebitis of the internal jugular vein and the formation of emboli. Management of tonsillitis is by encouraging the patient to take up plenty of fluid along with analgesics like paracetamol are prescribed to bring down the fever and to reduce the local pain, antibiotic therapy is advised too in which medication such as penicillin, ampicillin, or amoxicillin form the main stream of medication [5]. The tonsillectomy procedure is considered to be done in the case of recurrent tonsillitis which occurs frequently over a period of time, obstructive sleep apnoea, Eagle's syndrome, asymmetric enlargement of the tonsil, tumour involving palatine tonsil [6].

## Review

Lemierre’s syndrome is also known by different terminologies such as necrobacillosis or postanginal septicemia [7]. It starts as an oropharyngeal infection that broadens to give rise to the involvement of lateral pharyngeal spaces of the neck with thrombophlebitis of the internal jugular vein (IJV) and results in multiple emboli which accompanies formation of abscesses in the lungs and the joints. In 1900 physicians Courmount and Cade for the first time identified a condition as infection of the oropharynx which was caused by anaerobic bacteria leading to the formation of infective pulmonary infarcts after attaining intravascular entry. Later on, Goldman and Mosher in the years 1917 and 1920 and Fränkel in 1925 explained the conversion of simple infection of tonsils to thrombophlebitis of the tonsillar veins, along with the formation of a septic thrombus in the Internal jugular vein and its putrefaction into multiple septic emboli [8,9]. But even after appreciable work on this condition they were not able to find out the agent that gave rise to this deadly condition.

In 1936, Andre Lemierre, who was a professor by profession, arrived. Lemierre was a French microbiologist. He worked and elaborated on 20 cases of "postanginal anaerobic septicemia" [10]. Lemierre ascertained congruence of all the findings of his 20 cases that he worked on and got them published [11]. He individually went through the different stages of diseases in those cases and presented with results separately according to his prototype, confirming the microbiological organism liable for the occurrence of such a life-threatening condition as an anaerobic Gram-negative microorganism of rod shape named Bacillus funduliformis which got its name as Fusobacterium necrophorum later on. Lemierre also depicted the advancement of the condition from a local infection of tonsils of suppurative nature to the formation of septic emboli at distance from the local septic thrombophlebitis.

Today, it is considered an occasional disease, but it can't be denied that it is a life-threatening phenomenon. Before the advent of antibiotics, this disease had an extremely stormy and disastrous outcome with rapid progression only during the time of two weeks. Due to the entrance of antibiotics as a treatment, the rate of mortality has gone down to just 5%. About only 10% of the patients contact permanent consequences which constituted the exchange of valves due to endocarditis, meningitis abscess inside the cerebrum, and osteomyelitis [12]. Because of the arrival of antibiotic therapy Lemierre's syndrome has been named a "forgotten disease" [13].

Microbiology

In 90% of cases, the organism involved as the causative microorganism was Fusobacterium necrophorum, which is strictly an anaerobic bacteria that stains negative on gram stain and doesn't form spores. It is a part of the normal flora of the body and is present in the gastrointestinal tract of humans and animals. The pathogenic mechanism of F. necrophorum is complex and not well defined. Various factors that make it virulent are leukotoxin, endotoxin, and hemagglutinin [14]. Other microorganisms that can be linked as the responsible agent are groups A, B, and C Streptococcus, Staphylococcus aureus which is methicillin-resistant Enterococcus, Proteus mirabilis, Klebsiella pneumoniae, Pseudomonas aeruginosa, Bacteroides, Eikenella corrodens, Leptotrichia buccalis, Porphyromonas (asaccharolytica, endodontalis), Arcanobacterium haemolyticum or Prevotella bivia [15]. The following table (Table 1) gives information about the characteristics of the bacteria associated with Lemierre's syndrome; for example Staphylococcus that is associated in some cases is a facultative anaerobe. 

**Table 1:** Microorganisms associated with Lemierre's Syndrome

MICROORGANISM [16]	AEROBE OR ANAEROBE	GRAM POSITIVE OR GRAM NEGATIVE
Fusobacterium necrophorum	Anaerobe	Gram negative
Staphylococcus	Facultative anaerobe	Gram positive
Streptococcus	Facultative anaerobe	Gram positive
Klebsiella pneumoniae	Facultative anaerobe	Gram negative
Pseudomonas aeruginosa	Obligate aerobe	Gram negative
Leptotricha buccalis	Obligate anaerobe	Gram negative

Risk factors

Risk factors that can be attributed to this condition and which can give a strong hint about the development of such a grave disease are constant fever, history of infection of sinuses or infection of tonsils, tonsillitis or pharyngitis or history of any tumour pertaining to head, neck, throat region or the existence of any co-morbidity such as diabetes mellitus. Another strong risk factor is poor oral hygiene with multiple dental caries. The existence of any pathology renders the person to compromise his immunity [17].

Pathophysiology

The advancement of a local infection into intravascular space lacks evidence. However various theories have been reported and according to them the infection spreads through veins of the tonsils and peritonsillar veins but other vessels that can be affected in the pathogenesis are external jugular veins or facial veins. It was also observed that there existed explicit development involving the fascial plane and along the lymphatics. F. necrophorum is successful in causing infection and is able to invade as a result of various virulence factors associated with it. F. necrophorum sticks to cells of the epithelium and gets access to the tissues by the formation of plasmin which is a fibrinolytic enzyme. It develops a thrombus due to hemagglutinin due to which the platelets aggregate [18]. With the help of factor H and also by a protein that results in the stoppage of movement of white blood cells (WBCs) to the focal point of infection Fusobacterium, has the potential of deceiving the innate type of immunity of human beings. Under the presence of heparinase embolization, septic thrombi occur. Improper functioning of the endothelium, which is because of various virulence factors of Fusobacterium, results in the occurrence of thrombosis [19]. The discharge of infected emboli into the systemic circulation is because there occurs extensive propagation of the causative agent into various organs such as the spleen, liver, kidney, bones, muscles, and lungs. It can also cause spread to the central nervous system (CNS) resulting in the formation of abscesses and thrombus inside the cavernous sinus [9].

Clinical manifestation

The clinical manifestation of Lemierre's is studied under three phases [11]. Infectious involvement of oropharynx - this involves oropharynx infection which is followed by febrile illness and rigours occurring after four to seven days after the original infection. Patients also complain of dysphagia, photophobia, tonsillitis, pharyngitis, dyspnoea, loss of appetite or feeling of nausea can also be reported. Thrombophlebitis of the internal jugular vein due to the spread of infection in the parapharyngeal space of the neck, this is clinically reported as the presence of tenderness and swelling over the neck region, the existence of these signs give a hint about the progression of the disease out of the throat in the adjacent tissues. A separated area over or parallel to sternocleidomastoid muscle can present with a separate area of induration, oedema, and erythema [20]. Cord sign is also noted in 25% to 40% of cases which also gives hints about internal jugular vein thrombosis. It is identified by swelling and tenderness over one side at the mandibular angle. Formation of emboli that are infective at distant sites - septic emboli are shaped due to the result of thrombus formation. This happens when the cervical veins are infected [11]. The organ that is primarily affected by this spread is the lungs. It is presented in the patient by the complaints of a developing cough that is productive in nature. Pain in the chest, contaminated breath, along with the appearance of blood in the vomit is known as haemoptysis [21]. Other organs that can be involved are joints such as the shoulder joint, and knee joint with the clinical representation as arthralgias and sometimes even as osteomyelitis. When the brain is the affected organ it presents as VI and XII nerve palsies. Other organs that can be affected are the heart, soft tissues and kidneys. Liver involvement is clinically reported as jaundice or hepatomegaly. In rare cases the disease maybe complicated with the formation of liver abscess [22]. Septic shock is reported in approximately 7% of patients. Table 2 sheds light upon multiple throat and ear cases linked with Fusobacterium necrophorum. 

**Table 2:** Ear and throat cases associated with Fusobacterium necrophorom

Parameter [23]	Ear associated cases	Throat associated cases
Number of Cases	32	179
Age range	2 month- 51 years	8 month-63 years
% of cases with- Positive blood culture	81	89
Jugular venous thrombosis	19	48
Sinus thrombosis	25	2

Diagnosis 

The three pillars of the symptoms that help in the diagnose the case of Lemierre's are a recent history of oropharyngeal infection, thrombophlebitis of the internal jugular vein or of any vein of the head or neck region, and isolation of Fusobacterium necrophorum from anaerobic blood cultures [24]. The tests that can be employed in the detection of the syndrome are blood tests; various blood tests are of importance like C-reactive protein (CRP), which is useful for shedding light on the presence of inflammation inside the body [25]. Erythrocyte sedimentation rate (ESR) also tells about inflammation. A number of other abnormalities can be accessed with the help of routine blood examination such as leukopenia or even leukocytosis, increased levels of liver enzymes, elevated bilirubin, thrombocytopenia, creatininemia, and abundance in lactates. Coagulation studies should also be performed. Blood cultures should be performed over two to seven days that might show the occurrence of Fusobacterium but in many cases, the cultures are negative and don't show the bacteria owing to the problems in culturing by anaerobic procedures. By culturing the bacteria on the anaerobic media, a gram-negative bacteria that shows rod shape of different lengths which has circular ends and also shows twisting and swelling of the bacterial cell body [26].

Imaging methods form the cornerstone of the diagnosis of the condition. Confirmation of thrombus in the internal jugular vein appears to be the proof that shed the light on the diagnosis of Lemierre's syndrome. Computed tomography (CT) scans can be made use of for the assessment of the tender tissues of the body revealing swelling and inflammation. It includes the usage of a number of X-ray images interpreted by the machine and is changed to give rise to a multidimensional figure [27]. CT scans are useful for the doctor to access the location of the infection. It is considered the investigation of the choice [28]. It is also of great importance to evaluate for the presence of septic thrombosis of the various veins involved be it the internal jugular vein or other chest or neck vein this can be done by CT neck with contrast. There is advantage of clear visualization with help of a CT scan but there is an increased amount of exposure to the radiations which in turn can be harmful to the patient [29]. Ultrasound Doppler allows a clear image of the blood clots formed around the jugular veins. It is an extremely fast and non-invasive procedure. Though It is considered as a less sensitive method of assessment because the newly formed thrombi present with very little echogenicity, another drawback of this method is that it can be used only for the evaluation of the the supraclavicular tissues [9,29]. Ultrasound doppler is the technique which allows the evaluation of the rate of flow of blood inside the blood vessels by emitting waves of sound which are of increased frequency on the erythrocytes [30]. MRI It is a very sensitive modality for the diagnosis of the syndrome for ruling out if any thrombi present along and inside the internal jugular vein and especially when osteomyelitis is suspected [21], though the increased cost of the test makes it less approachable. A chest radiograph is useful as the lungs are the oragns that are the most affected by the distant septic emboli. It should be used to check for the presence of other complications which can be abscess formation or complaints of emphysema. Standard retrograde venography isn't performed nowadays for the purpose of diagnosis as it is a very intrusive procedure [31].

Treatment

As it is an infective condition antibiotics play a predominant role in the treatment of Lemierre's syndrome [11,15]. The antibiotics that can be used are penicillins such as carbapenem and piperacillin which were extensively used. It was used alone as monotherapy or along with metronidazole which proved to be successful in treating the disease. In patients with penicillin resistance or deficiency treating with the help of penicillin due to the production of beta-lactamase by the causative agent Fusobacterium necrophorum, beta-lactamase resistant beta-lactam antibiotic forms the fundamental treatment technique. Substitute therapy includes clindamycin and metronidazole for the cases presenting with an allergy to the beta-lactam medication. Metronidazole is useful as it has good seepage with the tissues along with good bioavailability by the oral route of administration. It is developed from nitroimidazole which is active against protozoans and bacteria. It is easily taken up by the bacteria that are strict anaerobes [32]. In the majority of the cases, the antibiotic regime is followed for approximately six weeks.

The concept of usage of anticoagulants in the treatment is not very well established and many arguments still persist over its use. The important role of anticoagulants is to stop the spread of thrombi throughout the body, especially inside the central nervous system including the sinuses. It helps in making the clots weaken and also stops the formation of the new thrombus [33]. It can be needed or adopted in cases of patients with a high burden of clot or thrombi formation, when there is a cerebral involvement of cavernous sinus by the thrombi, or within 72 hours when the patient fails to recover with appropriate antibiotic therapy. Low molecular weight heparin and also warfarin is used for anticoagulant therapy [34]. Use of anticoagulants is linked with a great risk of hemorrhagic complications. The course is followed for three months and close follow-up is needed for these patients. Surgical management by incision and drainage is employed in the case of complications due to the metastatic transfer of septic emboli [35] involving mediastinum and cerebrum and causing abscesses inside the organs such as the liver, epidura and neck. In a minority of the cases that present with extreme complications, the internal jugular vein is tied by surgical methods.

## Conclusions

Lemeirre's syndrome is hence an undeniably crucial and lethal complication of acute tonsillitis which can go undiagnosed in many cases. Acute tonsillitis is a commonly found disease in school-going children with the presenting complaints of pharyngitis, tonsils are red and swollen, fever and also jugulodigastric lymph nodes are expanded and grown in size. The clinical features which are associated with the condition are fever, rigors, and septic thrombi, which arise due to the rise of internal jugular vein thrombophlebitis which is the main presenting feature of the syndrome. The thrombi can be widespread inside the body and can affect various other organs of the body, lungs being the most commonly affected, the most commonly found organism is Fusobacterium necrophorum. Without proper treatment with antibiotics, this disease possessed a great threat to mankind and was considered a serious complication. In this article, we went through the various clinical manifestations that are linked with this condition. The crucial enzymes that are secreted by this bacterium are plasmin and heparinase which makes this bacterium invasive and also helps in escaping human immunity. Prompt and relevant diagnosis using appropriate blood cultures and imaging modalities that include CT Scan and MRI form the important step to check up on the location of the formation and spread of thrombi. Early treatment with antibiotics such as penicillins, metronidazole and beta-lactamase and in some cases with anticoagulants proves to be useful and necessary measures to cure the progression of the disease.
